# Fertilization, Oocyte Activation, Calcium Release and Epigenetic Remodelling: Lessons From Cancer Models

**DOI:** 10.3389/fcell.2022.781953

**Published:** 2022-03-04

**Authors:** Areez Shafqat, Junaid Kashir, Sulaiman Alsalameh, Khaled Alkattan, Ahmed Yaqinuddin

**Affiliations:** ^1^ College of Medicine, Alfaisal University, Riyadh, Saudi Arabia; ^2^ Department of Comparative Medicine, King Faisal Specialist Hospital and Research Center, Riyadh, Saudi Arabia

**Keywords:** oocyte activation, DNA methylation, calcium, cancer, fertlization

## Abstract

Oocyte activation deficiency (OAD) is the basis of Total Fertilisation Failure (TFF) and is attributed to mutations in the PLCζ gene—termed male factor infertility. This derives abnormal Ca^2+^ oscillations and could be the main cause of primary disruptions in the gene expression of Ca^2+^-related proteins. Epigenetic mechanisms are universally accepted as key regulators of gene expression. However, epigenetic dysregulations have not been considered as potential mechanisms of oocyte-borne OAD. Herein, we discuss changes in the DNA methylome during oogenesis and embryogenesis. We further highlight key pathways comprising the oocyte Ca^2+^ toolkit, which could be targets of epigenetic alterations, especially aberrations in DNA methylation. Considering that the vast majority of epigenetic modifications examined during fertilization revolve around alterations in DNA methylation, we aim in this article to associate Ca^2+^-specific mechanisms with these alterations. To strengthen this perspective, we bring evidence from cancer research on the intricate link between DNA methylation and Ca^2+^ signaling as cancer research has examined such questions in a lot more detail. From a therapeutic standpoint, if our hypothesis is proven to be correct, this will explain the cause of TFF in idiopathic cases and will open doors for novel therapeutic targets.

## Introduction

Fertilization encompasses the fusion of sperm and oocyte membranes, pro-nuclei formation, and the initiation of early embryogenesis. Following membrane fusion, the crucial trigger mediating downstream events is oocyte activation, defined by cortical granule exocytosis, resumption of meiosis II, and the induction of early embryogenesis (Kashir et al., 2010). Calcium (Ca^2+^) is the principal mediator of mammalian oocyte activation, displaying long-lasting oscillations from membrane fusion to pronuclei formation to kickstart signaling pathways culminating in oocyte activation (Kashir et al., 2010; Saleh et al., 2020). The trigger of Ca^2+^ oscillations is phospholipase ζ (PLCζ)—a sperm-specific PLC isoform—which produces inositol-1,4,5-triphosphate (IP_3_) via hydrolysis of phosphatidylinositol-4,5-biphosphate (PIP_2_) (Saunders et al., 2002). IP_3_ binds to IP_3_Rs on the endoplasmic reticulum (ER) to elicit Ca^2+^ release from internal stores.

The discovery of PLCζ and Ca^2+^ oscillations have revolutionized the world of assisted reproductive technology (ART) in treating infertility, a misfortune affecting ∼15% of couples worldwide. Firstly, intracytoplasmic sperm injection (ICSI) has proven instrumental as an alternative to conventional *in vitro* fertilization (IVF). ICSI circumvents the numerous *in vivo* barriers to fertilization by introducing sperm, and thereby PLCζ, directly into the ooplasm. However, partial or total fertilization failure (TFF) affects ∼1–5% of ICSI procedures where seemingly normal sperm and eggs exhibit failed fertilization (Yeste et al., 2016). Oocyte activation deficiency (OAD) is considered the main cause of TFF and is attributed to mutations in the PLCζ gene—termed male factor infertility [readers are referred to (Kashir, 2020; Zafar et al., 2021) for more detailed reviews]. To counter this, artificial oocyte activators (OAA), such as calcium ionophores (ionomycin and calcimycin), have been produced to induce Ca^2+^ transients in oocytes. But these molecules elicit a solitary [Ca^2+^] rise rather than the repetitive oscillations seen *in vivo*, which negatively impacts embryogenesis (Yeste et al., 2017). Consequently, developing exogenous PLCζ to trigger oscillations better approximating the *in vivo* fertilization is a focus of ongoing research (Kashir et al., 2010; Kashir et al., 2011).

However, although a large proportion of TFF cases are attributed to male factor infertility, approximately 26% of TFF cases are considered idiopathic (Yeste et al., 2016). This has significantly hindered the efficacy of ART. Indeed, ∼1—5% of ICSI procedures fail, success rates per cycle of ICSI remain a meager ∼27%, and global pregnancy rates and live births following ART is ∼40% (Kashir, 2020). Mechanistic studies behind these idiopathic cases of TFF are crucial in developing novel diagnostic and therapeutic procedures to improve ART efficacy. To this end, other potential SOAFs, such as PAWP, have been put forward as alternatives to PLCζ with mutations in them manifesting as TFF. However, this was proven unlikely, as knockout (KO) mice for the gene encoding PAWP (gene name *Wbp2nl*) showed no demonstrable defect in oocyte-activating ability (Satouh, 2015).

Even though male factor infertility accounts for a substantial percentage of TFF cases, the role of potential oocyte factors should not be dismissed; discussion on this topic, however, is limited. It would be prudent now to appreciate the dependence of PLCζ on various ooplasmic proteins. Mutations affecting these proteins could cause an oocyte-borne OAD. Even if oocyte activation is achieved, the atypical Ca^2+^ profile could have deleterious effects on embryonic development. Indeed, too few oscillations compromise the embryo’s ability to implant, whereas an amplified Ca^2+^ response impairs post-implantation development (Ozil et al., 2006). This relates to Ca^2+^ oscillations affecting gene expression (Ozil et al., 2006). Consistently, analysis of TFF metaphase II (MII) oocytes relative to controls revealed substantial differences in gene expression profiles of TFF oocytes, with genes involved in meiosis, cell growth, and apoptosis being disproportionately affected (Gasca et al., 2008).

Abnormal Ca^2+^ oscillations alternatively could derive from primary disruptions in the gene expression of Ca^2+^-related proteins. Epigenetic mechanisms are universally accepted as key regulators of gene expression and are known to regulate Ca^2+^ signaling genes. Yet, epigenetic dysregulations have not been considered as potential mechanisms of oocyte-borne OAD. In this manuscript, we discuss epigenetic programs during oogenesis and embryogenesis, emphasizing DNA methylation; since most of the epigenetic programming events studied during fertilization typically revolve around changes in DNA methylation profiles, we feel it appropriate to focus on this particular epigenetic process and uncover the Ca^2+^-specific mechanisms underlying DNA methylation alterations. Lastly, we present evidence from cancer research as a model which has investigated these questions in great detail and confirmed the intricate link between DNA methylation and Ca^2+^ signaling in somatic cells, further warranting an investigation into this relationship in oocytes. If this hypothesis proves favorable, new mechanisms to explain supposedly idiopathic cases of TFF could emerge alongside novel therapeutic targets.

## The Role of DNA Methylation During Oocyte Development and Early Embryogenesis

In short, epigenetic modifications regulate gene expression without altering nucleotide sequence. Epigenetic modulations include DNA methylation, histone tail post-translational modifications (PTMs), and non-coding RNAs (ncRNAs) (Le Blévec et al., 2020; Zafar et al., 2021). DNA methylation and histone PTMs regulate chromatin accessibility and packaging to control the binding of transcription factors to either facilitate or repress gene expression. On the other hand, ncRNAs are strong post-transcriptional regulators, targeting the 3′ untranslated regions (3′-UTRs) of specified mRNA molecules to induce their degradation (Le Blévec et al., 2020; Zafar et al., 2021).

While multiple epigenetic mechanisms are involved in determining the fate of gene expression profiles, the predominant player almost exclusively studied in the context of calcium release at fertilization has been methylation/demethylation of maternal and paternal DNA. This is also perhaps due to the consideration that in most mammals calcium oscillations persist for about 2–4 h post-gamete fusion, after which they cease around pronuclear formation. Additionally, while there are of course certain processes that could be influenced by the specific patterns of calcium release at fertilization, such processes are almost unique to fertilization and as such cannot be investigated in model systems such as cancer. One such example is sperm head decondensation postfertilization, which involves a protamine-to-histone change in DNA composition. During spermatogenesis, sperm nuclei are remodeled to incorporate protamine, which are small arginine-rich nuclear proteins that allow strong DNA binding to form highly stable and compacted DNA (Sassone-Corsi, 2002; Ward, 2010; Ribas-Maynou et al., 2021). However, 10–15% of the human sperm genome (∼1% in mice) remains histone-bound, suggesting that histones facilitate post-translational modifications that are transmitted to the early zygote and persist during pre-implantation embryogenesis (Hales et al., 2011). Indeed, histones play a key role in protamine transition and chromatin reorganization during spermatogenesis. It is thought that profiles of calcium release exert a significant role in the rate and quantity of protamine-to-histone transition (McLay et al., 2002), but this still requires further investigation.

### Mechanisms Behind DNA Methylation

DNA methylation involves covalent modification of DNA, in which a methyl group from S-adenosylmethionine (SAM) is donated to the carbon-5 of cytosine, forming 5-methylcytosine (5-mC) in a Cytosine-Guanine (CpG) dinucleotide context. The enzyme catalyzing DNA methylation are DNA methyltransferases (DMNTs), whereas methylation is erased by ten-eleven translocases (TETs) (Ito et al., 2011). Although CpG dinucleotides across the genome are methylated, CpG clusters termed CG islands—(CGIs) associated with gene promoters and enhancers (i.e., regulatory regions)—are constitutively unmethylated (Deaton and Bird, 2011). Overall, DNA methylation constitutes a repressive epigenetic mark.

### Epigenetic Reprogramming During Oogenesis & Preimplantation Embryogenesis

In sperm, DNA methylation is initiated prenatally and completed before puberty. In contrast, oocytes before puberty are practically unmethylated. Methylation in oocytes is initiated in the cohort of oocytes recruited at the beginning of each ovarian cycle to undergo ovulation. Methylation proceeds with follicular growth from the primary to the preantral and antral stages when methylation is completed (Sendžikaitė and Kelsey, 2019; Barberet et al., 2020). The mammalian oocyte methylome is established in a transcription-dependent manner, leaving non-transcribing genes and intergenic areas hypomethylated, resulting in a bimodal and clustered methylome consisting of hyper- and hypomethylated domains (Sendžikaitė and Kelsey, 2019).

Postfertilization, both paternal and maternal DNA is demethylated via TETs. Paternal DNA is demethylated actively and rapidly, unlike the passive demethylation characteristic of maternal DNA. This event is essential to abolish gamete identity and confer cells of the growing embryo with pluripotent potential (Cantone and Fisher, 2013; Sendžikaitė and Kelsey, 2019). Following implantation, DNA methylation is regained and proceeds in a lineage-specific pattern. Primordial germ cells (PGCs) subsequently experience a second wave of DNA demethylation to completely abolish parental epigenomes, which eventually are regained in a gender-dependent manner (Sendžikaitė and Kelsey, 2019).

Some regions of parental DNA evade demethylation during embryogenesis and are termed differentially methylated regions (DMRs). Most DMRs comprise imprinted loci which feature monoallelic expression depending on the parental origin of the allele (Tucci et al., 2019). Imprinted loci contain a cluster of genes whose expression depends on the DNA methylation status of an imprinting control region (ICR). ICRs of imprinted loci contain a CG-rich sequence recognized by the KRAB zinc-finger protein 57, which subsequently recruits a set of regulatory proteins to protect these sites (Tucci et al., 2019). Therefore, epigenetic marks on imprinted loci are inherited and maintained throughout life, only being erased in PGCs.

Other epigenetic modifications such as histone-PTMs also exhibit reprogramming during early embryogenesis. Histones are closely associated with DNA to form nucleosomes that contain two each of histones H2A, H2B, H3, and H4. Each histone has N-terminal tails with amino acid residues that serve as potential sites of covalent modifications such as acetylation, methylation, or phosphorylation. Histone PTMs are found at distinct loci within the genome and serve to alter the spatial arrangement of nucleosomes and modulate chromatin structure to control binding of transcription factors, thereby regulating gene expression (Kouzarides, 2007). More layers of complexity are added by the same covalent modifications at distinct loci having varying effects; for example, tri-methylation at Lysine 9 of H3 (H3K9me3) silences gene expression, whereas methylation of lysine 4 of H3 (H3K4me) is a marker of active transcription. During early embryogenesis, a major resetting of histone PTMs occurs with clear asymmetry between paternal and maternal epigenomes. For instance, in mice, mature oocyte non-canonical H3K4me3 marks are distributed broadly in both promoters and distal regions and are inherited by the embryo and persist until zygotic genome activation (ZGA), when they are removed at the late two-cell stage. In contrast, sperm H3k3me3 and H3k27me3 are quickly removed after fertilization (Zhang et al., 2016). However, recent advances in the field have allowed the detection of histone PTM reprogramming in human pre-implantation embryogenesis (Skene and Henikoff, 2017), revealing important differences between mice and humans in this regard (Xia et al., 2019). Indeed, in human oocytes, H3K4me3 features sharp peaks at promoters, and weaker distal H3K4me3 marks are seen in pre-ZGA embryos (Xia et al., 2019). However, the function of these histone marks in humans remains to be identified.

A non-coding RNA (ncRNA) is not translated into a protein. 90% of the eukaryotic genome is transcribed, but mRNA accounts for only 1–2% of total RNA (Ponting and Grant Belgard, 2010). The remaining ∼98% are ncRNAs, which are further divided into “housekeeping” RNAs (including transfer RNA and ribosomal RNA required for translation) and “regulatory RNAs”, which are divided according to size into small ncRNAs (sncRNAs), which include PIWI-interacting RNAs (piRNAs), small interfering RNAs (siRNAs), and micro-RNAs (miRNAs), and long ncRNAs (lncRNAs) (Deniz and Erman, 2016). Recently, knowledge on the role of ncRNAs in regulating different aspects of biology led to the discovery of their role in gametogenesis and embryogenesis, which is comprehensively reviewed here (Pauli et al., 2011). piRNAs are essential for spermatogenesis and fertility in mice, presumably by preventing the accumulation of transposons (Fu and Wang, 2014). Furthermore, miRNAs and siRNAs are involved in heterochromatin formation, transcriptional silencing, and DNA repair during spermatogenesis (Hilz et al., 2016). In contrast, sncRNAs are non-essential for oogenesis and early embryogenesis pre-ZGA. However, ncRNAs are essential in embryogenesis post-ZGA, as the zygotic genome expresses miRNAs to degrade maternal mRNAs transcribed during oogenesis. Additionally, regulatory ncRNAs are responsible for maintaining pluripotency of embryonic stem cells and their differentiation, germ layer specific, cell fate specification, and morphogenesis, as is comprehensively reviewed here (Pauli et al., 2011).

However, it remains that in a developmental context, DNA methylation reprogramming is best characterized (Zhu et al., 2021). Furthermore, demethylation processes ensue immediately following fertilization; the sperm genome is fully demethylated before the first cell division, whereas the passive demethylation characterizing the maternal epigenome begins and continues over the subsequent cell divisions (Seisenberger et al., 2013). Consistent with this temporal association between Ca^2+^ oscillations and DNA demethylation, altered DNA methylation patterns are associated with different oocyte activation protocols utilized in ART. Indeed, the long-term epigenetic alterations observed in ART involve environmental disturbances experienced by *in vitro* embryos, and DNA methylation changes are largely implicated in this setting (Zhu et al., 2021). In contrast, histone PTM reprogramming begins later in preimplantation embryogenesis, decreasing the likelihood of a potential interplay with fertilization-associated Ca^2+^ dynamics; the same applies to ncRNAs. Therefore, this perspective highlights the potential interplay between gene expression and Ca^2+^ dynamics at fertilization through Ca^2+^-induced changes in DNA methylation patterns.

### Could an Altered Ca^2+^ Oscillation Profile Disrupt DNA Methylation Programs?

Abnormal Ca^2+^ oscillations postfertilization are confirmed to yield suboptimal developmental outcomes. The mechanism behind this, however, remains to be elucidated. What is known is that abnormal postfertilization Ca^2+^ profiles disrupt gene expression, as evidenced by experimental manipulation of Ca^2+^ oscillations modifying the blastocyst transcriptome (Stein et al., 2020). Calcium signaling parameters, particularly amplitude, are important determinants of postfertilization development (Ozil and Huneau, 2001). Dampened oscillations jeopardize preimplantation development, with genes of RNA processing and polymerase-II transcription particularly affected (Ozil et al., 2006). In contrast, hyperstimulation of Ca^2+^ oscillations compromises postimplantation development by lowering blastocyst developmental competence. In this context, genes involved in metabolism were preferentially involved (Ozil et al., 2006). A study assessing strontium-induced parthenogenetic oocyte activation—characterized by repetitive Ca^2+^ oscillations—exhibited higher expression of genes encoding proteins involved in cell proliferation, cell adhesion, and ion transport. In contrast, microarray analysis of gene expression profiles of embryos retrieved from oocytes activated in a Ca^2+^-devoid manner exhibited higher expression of genes of the cell cycle, apoptosis, and cell differentiation (Rogers et al., 2006). In addition, these embryos showed decreased developmental rates to the blastocyst stage and a reduced inner cell mass, highlighting the importance of Ca^2+^ in proper cell division (Rogers et al., 2006).

In short, postfertilization Ca^2+^ profiles are confirmed to influence the nature of subsequent cell divisions and embryonic development, with this effect possibly being mediated through alterations in embryo gene expression. Simultaneously, major epigenetic reprogramming characterized by parental DNA demethylation is being established; such programs ensue immediately following Ca^2+^ oscillations. Therefore, could abnormal Ca^2+^ oscillations disrupt these programs? Future work should address this question by evaluating the epigenetic profiles of affected genes. If such mechanisms are at play, and since epigenetic modifications are reversible, efforts to correct dysregulation of the oocyte DNA methylome could prove fruitful.

ART protocols are used globally to treat infertility. Current data regarding the frequency of congenital defects in children conceived through ART look reassuring. However, a small body of evidence associates certain procedures, including ICSI, with an increased risk of imprinting disorders, including Prader-Willi and Angelman syndrome (Palermo et al., 2009). This stems from studies revealing DNA methylation of certain genes and histones being impaired after ART [for more detail, review (Ciapa and Arnoult, 2011)]. The mechanism behind such disruptions, however, is currently unknown. Intriguingly, ICSI elicits Ca^2+^ oscillations deviating from the physiologic transients seen *in vivo* (Tesarik and Sousa, 1994; Kurokawa and Fissore, 2003). A study comparing the Ca^2+^ oscillatory pattern elicited by ICSI to normal IVF reported a longer duration of oscillations in the IVF group, with consequent reductions in cell numbers in the ICSI group and a concurrent slower hatching rate of ICSI-generated zygotes compared to IVF (Kurokawa and Fissore, 2003). Such oscillations, as highlighted above, affect gene expression profiles during embryonic development. Might the abnormal Ca^2+^ profile caused by ICSI be responsible for these epigenetic defects? Ca^2+^ oscillations regulating embryo gene expression and ICSI-induced atypical oscillations manifesting as epigenetic defects indicate a possible causal relationship between the two processes, meriting an in-depth investigation.

**FIGURE 1:**
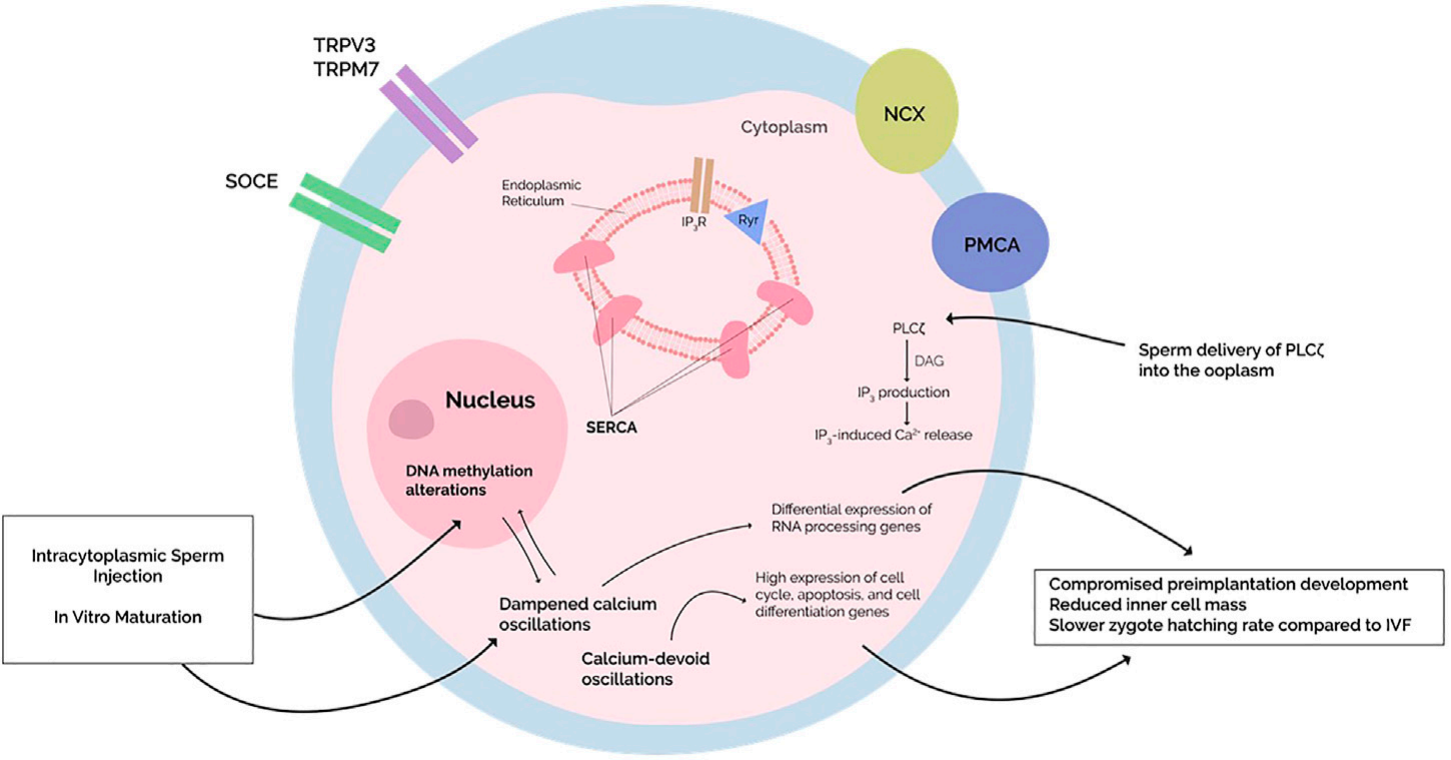
Dampening or absence of calcium oscillations is seen in intracytoplasmic sperm injection (ICSI) and cycloheximide-induced oocyte activation, respectively. This alters embryonic development by altering gene expression. The molecular mechanisms behind such changes, however, remain unknown. Since an increased risk of epigenetic defects is perceived in ISCI with simultaneous changes in DNA methylation patterns, perhaps the DNA methylation alterations and differential gene expression are due to the dampened oscillations since major epigenetic processes occur immediately after the cessation of calcium oscillations. Alternatively, primary dysregulations in the oocyte DNA methylome—as induced by ICSI or *in vitro* maturation—may affect calcium homeostasis, although further studies need to be conducted.

Calcium also leads to the production of reactive oxygen species (ROS). ROS generation plays a role in cell cycle resumption during both oocyte maturation and activation. During oocyte activation, physiologic Ca^2+^ transients elicit ROS levels sufficient to cause cell cycle resumption (Tiwari et al., 2017). However, in cases of abnormal Ca^2+^ profiles, overproduction of ROS exerts oxidative stress (OS) on oocytes, promoting apoptosis through intrinsic mitochondrial and extrinsic FAS receptor-ligand pathways (Tiwari et al., 2017). Furthermore, oxidative stress (OS) is a well-established modifier of the oocyte DNA methylome: guanine is most susceptible to OS, whereas oxidation of cytosine produces 5-hydroxymethylcytosine (5-hmC), a physiological prerequisite for DNA demethylation. In addition to initiating aberrant DNA demethylation, ROS can reduce the affinity of DMNTs for DNA [for more detailed descriptions refer to (Menezo et al., 2016)]. Hence, perhaps Ca^2+^ -dependent OS disrupts the oocyte DNA methylome, resulting in TFF (Figure 1). This would make sense of the aforementioned observation where TFF MII oocytes display altered expression profiles disproportionately involving genes encoding apoptosis-mediating proteins (Gasca et al., 2008).

## Calcium Toolkit and Signaling Molecules

PLCζ-induced Ca^2+^ oscillations activate numerous pathways concluding in cortical granule exocytosis, meiosis II resumption, and pronuclear formation. The initial event of oocyte activation is cortical granule exocytosis, characterized by the exocytosis of enzymes that modify the zona pellucida and prevent further sperm entry—blocking polyspermy. Substantial evidence currently exists implicating protein kinase C as the principal mediator of cortical granule exocytosis. Calcium transients activate PKCs which translocate to the oocyte cortex, phosphorylating myristoylated alanine-C-rich proteins (MARCKS). This disrupts the actin cytoskeleton, allowing for exocytosis of cortical granules to prevent polyspermy (Ducibella and Matson, 2007; Tsaadon, 2008).

Meiosis resumption and pronuclei formation follow cortical granule exocytosis. Metaphase II (MII) arrest is maintained by cytostatic factor (CSF), an inhibitor of anaphase-promoting complex (APC). Specifically, endogenous meiosis inhibitor (Emi2)—a subunit of CSF—inhibits APC. APC degrades cyclin B1 (CCNB1) of the CCNB1/cyclin-dependent kinase 1 (CDK1) complex, collectively termed maturation promoting factor (MPF). Therefore, by inhibiting APC, CSF maintains high levels of MPF to sustain MII arrest. Emi2 is degraded by polo-like kinase (Plk1), which itself requires a preceding phosphorylation step. Plk1 is phosphorylated—and activated—by calcium/calmodulin kinase II (CAMKII), an intracellular kinase activated by Ca^2+^ oscillations. Therefore, acute rises in intracellular Ca^2+^ activate CAMKII, leading to phosphorylation of Plk1 which degrades Emi2 subunit of CSF, thereby relieving APC from inhibition which then degrades tCCNB1 of the MPF complex. A second mechanism by which CAMKII promotes meiotic resumption is by phosphorylating and activating WEE1B. WEE1B, in turn, phosphorylates CDK1, leading to inhibition of MPF and meiosis resumption. Finally, the degradation of MPF triggers a decline in the activity of mitogen-activated protein kinase (MAPK), allowing pronuclei formation.

Therefore, CAMKII is the principal transducer of Ca^2+^ oscillations, which was confirmed by injection of CAMKII complementary RNA (cRNA) resulting in meiosis II resumption and pronuclear formation (Knott et al., 2006). CAMKII cRNA injection into eggs also yields developmental success rates similar to physiologic activation (Knott et al., 2006). More detailed descriptions of the molecular events of egg activation are found here (Wakai et al., 2011; Sanders and Swann, 2016).

### The Rise in Cytosolic Calcium ([Ca^2+^]_I_)

Just as the importance of Ca^2+^ oscillations has been emphasized, equally important are the transport proteins allowing these oscillations to occur. To support calcium signaling, somatic cells and oocytes possess a unique calcium toolkit that functions to maintain low cytosolic [calcium] ([Ca^2+^]_I_)—approximately 100 nM (Clapham, 2007). This calcium signaling toolkit comprises various ion channels, atpase pumps, and transporters on organelle membranes and the plasma membrane (PM). A complex interplay exists between these various components to generate cell- and stimulus-specific Ca^2+^ responses. A detailed review of the Ca^2+^ toolkit is found here (Carvacho et al., 2018).

Increases in [Ca^2+^]_I_ primarily result from Ca^2+^ release from internal stores through the activation of the inositol-1,4,5-triphosphate receptor (IP_3_R) and ryanodine receptor (RyR) on the endoplasmic reticulum membrane (ER). The IP_3_R, predominantly the IP_3_R_1_ isoform in oocytes, is essential to rises in [Ca^2+^]_I_: IP_3_R_1_ activation requires both Ca^2+^ and IP_3_, resulting in Ca^2+^ influx at low [Ca^2+^]_I_ and closure via negative feedback when [Ca^2+^]_I_ is elevated (Tosti, 2006; Kashir et al., 2014; Machaty et al., 2017). The RyR shares this pattern of activity.

Internal Ca^2+^ release is supported by extracellular influx. Store-operated calcium entry (SOCE), elicited by the depletion of [Ca^2+^]_ER_, occurs through an ER Ca^2+^ sensor stromal interaction molecule (STIM1) that recruits ORAI1 at PM-ER junctions to allow Ca^2+^ entry (Lewis, 1999; Carvacho et al., 2018). The voltage-gated T-type Ca^2+^ channel (Ca_v_3.1–3.3) and members of the transient receptor potential (TRP) family, specifically TRPV3 and TRPM7, also contribute to extracellular Ca^2+^ influx (Liu et al., 2011; Carvacho et al., 2013; Bernhardt et al., 2015).

### The Decline in Cytosolic [Ca^2+^]

To sustain oscillations and prevent cellular toxicity [Ca^2+^]_I_ must normalize and ER Ca^2+^ stores ([Ca^2+^]_ER_) replenished. To achieve this, sarcoplasmic reticulum Ca^2+^ ATPases (SERCA) and plasma membrane Ca^2+^ ATPases (PMCAs) pump calcium into the ER and extracellular space, respectively (Berridge et al., 2003). SERCA, specifically SERCA2b in oocytes, allows [Ca^2+^]_I_ and [Ca^2+^]_ER_ to display parallel but opposite oscillatory responses, where a rise in [Ca^2+^]_i_ is accompanied by a simultaneous decline in [Ca^2+^]_ER_. Sodium-calcium exchangers (NCX) also participate in the extrusion of Ca^2+^ into the extracellular milieu in rodent oocytes (Carroll, 2000).

The mitochondria aid SERCA and PMCA in buffering [Ca^2+^]_I_ by taking up Ca^2+^ (Rizzuto et al., 2000). Importantly, the rise in mitochondrial Ca^2+^ content sustains ATP levels in oocytes and eggs. Furthermore, inhibiting mitochondrial ATP production by oligomycin impairs refilling of [Ca^2+^]_ER_, indicating that Ca^2+^-induced ATP production during transients sustains SERCA activity (Wakai et al., 2013).

### Differential Expression of the Ca^2+^ Toolkit During Oocyte Development

Intriguingly, these Ca^2+^ channels are not expressed uniformly throughout oocyte development. Rather, each channel exhibits differential expression. SOCE- and Ca_v_3.1–3.3—mediated currents are maximal in GV oocytes and progressively diminish over the subsequent stages of oocyte maturation (Cheon et al., 2013; Bernhardt et al., 2015; Carvacho et al., 2018). In contrast, TRPV3 expression is negligible at maturation and progressively increases to maximal levels in MII oocytes (Carvacho et al., 2018). The mechanisms underlying this differential expression are unknown. However, since this process coincides with the insidious establishment of the oocyte DNA methylome, investigations into a potential interplay are warranted.

### Disruptions in the Oocyte DNA Methylome Altering Ca^2+^ Oscillations

**FIGURE 2:**
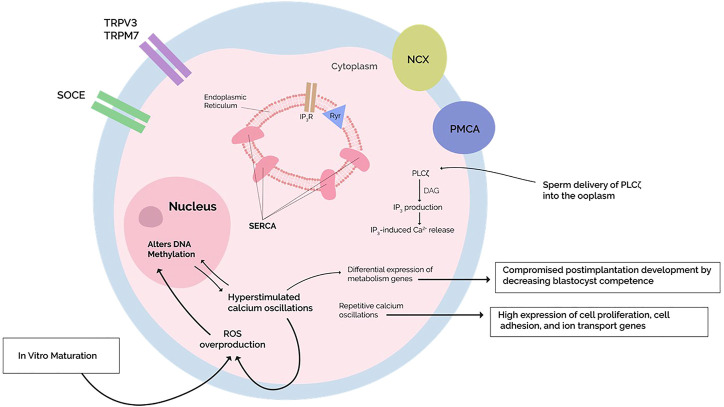
Repetitive calcium oscillations approximating *in vivo* fertilization feature a differential gene expression with high expression of cell cycle, cell adhesion, and ion transport genes. On the other hand, hyperstimulation of calcium oscillations compromises postimplantation development by lowering blastocyst competence with high expression of metabolism-related genes. Simultaneously, the Hyperstimulated calcium transients increase the production of reactive oxygen species (ROS), which are known to induce changes in DNA methylation through oxidative stress. The mechanisms underlying the observed differential expression are unknown, but when coupled with mounting evidence of calcium-dependent ROS generation altering the oocyte DNA methylome, perhaps these epigenetic alterations account for the differential expression. Studies investigating a potential causal relationship are warranted. *In vitro* maturation (IVM) protocols generate increased oxidative stress on the oocyte, altering DNA methylation programs during oocyte maturation—differential expression is indeed reported after IVM, but whether it also disrupts calcium homeostasis and what effects this has on postfertilization calcium oscillations remains investigational.

In short, various proteins maintain intracellular Ca^2+^ homeostasis, allowing postfertilization Ca^2+^ oscillations to occur. Abnormalities in such mechanisms negatively impact Ca^2+^ transients (Yeste et al., 2016). Research or clinical consideration of these scenarios, however, is rare. Accordingly, we propose that genes encoding Ca^2+^ transporters could be epigenetically silenced through DNA methylation, manifesting as abnormal Ca^2+^ profiles (Figure 2). Similar postulations have been made by other studies (Ciapa and Arnoult, 2011; Stein et al., 2020), although the connection between DNA methylation and Ca^2+^ oscillations has not been stated.

During *in vitro* maturation (IVM), oxidative phosphorylation in mitochondria may be disturbed along with the processing of genes pertaining to epigenetics and the cell cycle (Virant-Klun et al., 2018) [discussed comprehensively here (Zhang et al., 2021)]. A study compared the abundance of mRNA transcripts within IVM and *in vivo* matured oocytes, revealing substantial differences in polyadenylated mRNA transcript abundance of oocyte genes Mater and Zar1—both play important roles in postfertilization development—and gdf9, which is involved in conferring postfertilization developmental competence (Camargo et al., 2019). The changes in gene expression of these proteins may underpin the reduced developmental competence of IVM oocytes. Furthermore, the mRNA transcript abundance of heat shock proteins and peroxiredoxin is also reduced (Camargo et al., 2019). These proteins have antioxidant functions, protecting the oocyte from OS. Therefore, the reduced abundance of these genes may be associated with an increased OS on the oocyte. As discussed above, OS disrupts the oocyte DNA methylome (Camargo et al., 2019). This is one illustration of how the oocyte DNA methylome may get disrupted. But whether such disruptions involve Ca^2+−^homeostasis genes during oocyte development remains investigational. Although, the studies mentioned prior found no changes in the polyadenylated mRNA transcript abundance of such genes.

However, the *in vivo* matured oocytes were also stimulated by exogenous hormones FSH and LH in these experiments; these oocytes were not matured in natural conditions. The differences reported in this experiment might therefore not reflect the true extent of gene expression differences between *in vivo* and *in vitro* matured oocytes. Additionally, evidence from cancer research describes DNA methylation and Ca^2+^ homeostasis as being intricately linked in several somatic cells: studies have substantiated Ca^2+^ homeostasis disruptions as being predicated on alterations in the promoter methylation status of genes encoding vital Ca^2+^ proteins, thus leading to dysregulations in proper progression through the cell cycle (Izquierdo-Torres et al., 2020).

## Alterations in DNA Methylation and Calcium Homeostasis During Carcinogenesis

Fundamentally, carcinogenesis is characterized by dysregulation of the cell cycle, which is dependent on Ca^2+^ (Kim et al., 2020). Altered Ca^2+^ homeostasis underlies the acquisition of several hallmarks of cancer, including sustained proliferative signaling, replicative immortality, cell death resistance, angiogenesis induction, invasion and metastasis, and altered metabolism (Hanahan and Weinberg, 2011; Berridge, 2012; Izquierdo-Torres et al., 2020). Similarly, in the setting of fertilization, the egg is released from metaphase II arrest, which is triggered by Ca^2+^ by largely the same proteins. Therefore, the fertilization Ca^2+^ signal also controls cell cycle progression (Whitaker, 2008). Embryos derived from Ca^2+^-devoid fertilization consistently display a reduction in inner cell mass at the blastocyst stage (Rogers et al., 2006). CAMKII is the key protein ensuring proper cell cycle progression in both scenarios. The emergence of CAMKII in controlling cancer cell proliferation is confirmed by experiments pharmacologically inhibiting CAMKII to reduce tumor mass and proliferation (Wang et al., 2015).

Numerous studies have pointed out similarities between tumor and developmental biology. Preimplantation embryo cells undergo genome-wide reprogramming in the form of DNA demethylation, which confers pluripotent potential. This continues in primordial germ cells, which become undifferentiated and also display migratory capabilities (Fujimoto, 1977). When stringent vivo regulations are removed, such cells can divide indefinitely, i.e., display immortality. Furthermore, the embryo trophoblast exhibits invasive properties during implantation, characterized by invasion of the maternal uterine wall (Monk and Holding, 2001). *Cancer* cells are also undifferentiated, immortal, and invasive. In theory, these hallmarks of cancer may be the consequence of the re-expression of such developmental genes in an entirely inappropriate context (Manzo, 2019). Indeed, when transplanted into adult mice, embryonic cells give rise to tumors (Hogan, 1981). Several tumors accordingly feature a gene expression signature approximating a developing embryo, which is correlated with more aggressive phenotypes. A study evaluating the gene expression signature of 293 lung tumors revealed a reversion to a germ cell expression pattern in the most aggressive tumors (Rousseaux et al., 2013). Similarly, a report analyzing colorectal cancer genomic data implicated promoters of developmental genes as major targets of deregulations in their methylation status (An et al., 2015). Embryogenesis involves the precise regulation of several organized signaling pathways, which are then silenced in adult somatic cells. Examples of key developmental signaling pathways reactivated in cancer include the Wnt, Hedgehog, and Notch pathways (Aiello and Stanger, 2016). The reactivation of such pathways—by mutations or epigenetic alterations—is a hallmark feature of cancer, exemplifying the commonalities between cancer and developmental biology.

Epigenetic disruptions, including DNA methylation modifications, contribute to the oncogenic process, leading to aberrant gene activity. Both DNA hypermethylation and hypomethylation are detected in cancer cells: hypermethylation occurs on promoter regions of tumor suppressor genes (TSG), thus leading to gene silencing and tumor progression (Ehrlich, 2002; Locke et al., 2019). In contrast, hypomethylation occurs on DNA repeat sequences, promoting genome instability or oncogene activation.

The genes encoding the Ca^2+^ signaling toolkit have emerged as targets of aberrant DNA methylation, thus altering Ca^2+^ homeostasis to perpetuate the carcinogenic process. By rewiring Ca^2+^ signatures, cancer cells acquire a proliferative advantage compared to typical cells, allowing them to proliferate indefinitely amongst other cancer hallmarks. A study analyzing DNA methylation patterns in 12 cancer cell types showed Ca^2+^- toolkit genes—including NCX, CAMK, PMCA, PKC, IP_3_R—as being major targets of hypermethylation and downregulation (Wang et al., 2017). Likewise, alterations in expression and activity levels of SERCA, PMCA, TRPM7, ORAI/STIM1-mediated SOCE, and IP_3_R isoforms have been demonstrated in several cancer cell types [detailed reviews can be found here (Marchi and Pinton, 2016; Raynal et al., 2016; Izquierdo-Torres et al., 2020)]. Studies in pancreatic cancer have revealed significant rewiring of cancer cell Ca^2+^ signatures, with subsequent analysis on gene expression profiles implicating aberrant epigenetic programs as underlying mechanisms (Gregório et al., 2020; Kutschat et al., 2021). Similar findings are seen in hepatic cancer stem cells (CSCs), which display altered Ca^2+^ dynamics due to upregulation of the IP_3_R_2_ gene expression (Sun et al., 2019); whether or not these are due to epigenetic modulations are unknown. This study further implicated the altered Ca^2+^ profiles as underlying the self-renewal of CSCs, with IP_3_R_2_ knockdown consistently suppressing tumor formation (Sun et al., 2019). Because Ca^2+^ functions as a key second messenger, cancer cells—through a rewiring in their Ca^2+^ signaling machinery—withstand various adverse stimuli and exhibit unique cellular transcriptomes, leading to increased cell proliferation, invasion, and resistance to apoptosis. This rewiring takes place at the genetic level, with aberrant DNA methylations crucial to tumor instigation (Parkash and Asotra, 2010; Gregório et al., 2020).

Epigenetic changes are reversible: therapeutic correction of methylation status is correlated with a significantly more favorable prognosis (Kim et al., 2019; Meneses-Morales et al., 2019; Patergnani et al., 2020). For example, the SERCA3 gene (ATP2A3) exhibits hypermethylation and decreased expression in colon cancer: patients with low expression levels of SERCA3 displayed an average survival of 16.6 months, whereas patients with high expression levels survived 26.7 months (Meneses-Morales et al., 2019). This was consistent with pharmacologic administration of DMNT inhibitors (DMNTi) to induce SERCA3 expression, which decreased cell viability (Meneses-Morales et al., 2019). Collectively, pharmacologically targeting epigenetic-induced disturbances in Ca^2+^ signaling is currently seen as a potentially feasible and efficacious pharmacologic strategy to combat cancers. Nevertheless, in keeping with the present discussion, identifying Ca^2+^ channels, transporters, or signaling molecules in oocytes showing OAD and elucidating whether epigenetic dysregulations these findings would not only further our understanding of OAD and TFF but also could inform the identification of novel druggable targets to correct such ailments.

Ca^2+^ signaling in itself also modulates gene expression through various signal transduction pathways—the mechanism through which Ca^2+^ profiles regulate cell cycle progression [readers are referred to (Parkash and Asotra, 2010; Marchi and Pinton, 2016) for more detailed descriptions]. Briefly, several early cell-cycle mediating genes in G1 and phosphorylation status of retinoblastoma protein (Rb) in the G1/S transition phase are regulated by Ca^2+^. In addition, CaMK mediates physiologic cell cycle progression, as evidenced by inhibition of CaMK causing cell cycle arrest. Another Ca^2+^-dependent protein, calcineurin, mediates progression through the G1 and S phases through activating transcription factors CREB1 and NFAT, which upregulate gene expression of target genes inducing cell cycle progression (Parkash and Asotra, 2010; Cui et al., 2017). In turn, the activation of CaMK and calcineurin requires Ca^2+^ oscillations generated through the aforementioned Ca^2+^ toolkit. Therefore, perturbations in the Ca^2+^ signaling toolkit indirectly influence gene expression (Izquierdo-Torres et al., 2020). However, studies on Ca^2+^ profiles influencing epigenetic programs are limited.

To this end, a study described 11 pharmacologic agents, including cardiac glycosides, as triggering TSG reactivation—originally epigenetically silenced—in colon cancer (Cui et al., 2017). However, rather than directly modifying the epigenome, such drugs trigger CAMKII activity. Activation of CAMKII causes the release of methyl CpG binding protein from methylated promoters, inducing gene reactivation. Consistently, abolishing CAMKII activity blocked TSG reactivation (Cui et al., 2017). In short, drugs altering CAMKII activity indirectly reactivate epigenetically silenced TSGs via demethylation. Since CAMKII activity relies on Ca^2+^ profiles, perhaps targeting Ca^2+^ oscillation-generating channels and transporters represents a therapeutic pathway to reconstitute normal epigenetic patterns in cancer cells, albeit further research is required to address these issues.

In the context of oocyte activation deficiency, could aberrant Ca^2+^ responses—through CAMKII—lead to epigenetic modifications in genes involved in subsequent development, thus accounting for the reduced developmental success rates seen in oocytes displaying abnormal Ca^2+^ profiles? CAMKII mediates meiotic resumption during oocyte activation and affects preimplantation embryogenesis. Abnormal postfertilization Ca^2+^ oscillations-induced changes in CAMKII activity could therefore alter gene expression via epigenetic dysregulation, accounting for the impaired developmental competence of these eggs. At the same time, it is important to consider that epigenetic changes also target Ca^2+^ homeostasis. In this context, the antihypertensive drug hydralazine induces DNA demethylation of the SERCA2a gene, activating gene expression and thus leading to improved cardiac performance (Kao et al., 2011). Likewise, methamphetamine induces demethylation of the Ca_v_1.2 (L-type Ca^2+^ channel) gene in mice cardiomyocytes, leading to overexpression and a consequent hypercontractile state as evidenced by histopathologic examination showing contraction band necrosis (Koczor et al., 2015). Such discoveries should prompt investigations into possible pharmacologic interventions aimed at epigenome correction in cancer cells and oocytes.

## Conclusion and Future Directions

We investigated the potential link between Ca^2+^ signaling and homeostasis and DNA methylation alterations in oocytes, intending to elucidate a novel mechanism of OAD; one which involves Ca^2+^ homeostasis alterations secondarily disrupting the oocyte DNA methylome or vice versa. To strengthen our hypothesis, we discussed the well-established link between Ca^2+^ profiles and epigenome regulation in cancer cells, which given the similarities in cancer and developmental biology, constitutes a suitable model to study this process.

To this end, comparative studies evaluating the differential expression between normal oocytes and those showing OAD—and those showing TFF after ICSI or IVM—by employing microarrays would constitute a start. Using tiled DNA microarrays, potential causal DNA methylation changes can be identified. Subsequently, pathway enrichment analysis can identify families of genes targeted by such a process. If the Ca^2+^ signaling toolkit is involved, as is suggested by cancer research and drugs affecting the myocardium, then novel research directions and potential therapeutic and prophylactic strategies might, theoretically, result.

## Data Availability

The original contributions presented in the study are included in the article/Supplementary Material, further inquiries can be directed to the corresponding author.
